# Evaluation and Comparison of Force Degradation Levels Between Elastomeric Chains and Active Tiebacks With Elastic Modules During Individual Canine Retraction

**DOI:** 10.7759/cureus.69110

**Published:** 2024-09-10

**Authors:** Sanika M Thakur, Usha Shenoy, Ananya Hazare, Himija Karia, Pritam R Khorgade, Nivedita Nandeshwar, Sangeeta Bhattacharya

**Affiliations:** 1 Orthodontics and Dentofacial Orthopedics, Ranjeet Deshmukh Dental College and Research Centre, Nagpur, IND

**Keywords:** active tieback, degradation, elastomeric chain, force, modules

## Abstract

Introduction: Canine retraction is critical in orthodontic treatment, requiring precise force application. Elastomeric chains and active tiebacks are commonly used materials, but their efficacy in maintaining the force over time needs further evaluation. The study aimed to compare the rate of force degradation of elastomeric chains vs. active tiebacks during canine retraction over a duration of six weeks in patients undergoing fixed orthodontic therapy.

Methods: This study involved 20 patients, aged between 16 and 25 years. All the subjects were treated with the 0.018” McLaughlin-Bennett-Trevisi (MBT) prescription. Following the initial leveling and alignment, the canines were retracted on 0.016 x 0.022” stainless steel wire, using an elastomeric chain (ORMCO) on one side and an active tieback (ORMCO) on the contralateral side, each exerting a force of 150 g. Force values were measured using a tension gauge (TECLOCK; DT- 300) at intervals of 48 hours, two weeks, four weeks, and six weeks by a single observer and documented accordingly.

Results: The elastomeric chain and active tieback sides demonstrated substantial force degradation over six weeks, with a marked reduction of force at given intervals. However, no statistically significant difference in force degradation rates was observed between the two methods (p > 0.05), indicating similar force degradation patterns.

Conclusion: The elastomeric chain and active tieback methods demonstrated substantial force degradation over six weeks, with no statistically significant difference between the two. Thus, both materials can be effectively utilized for canine retraction in orthodontic treatment as they exhibit similar patterns of force degradation.

## Introduction

The fundamental goal of orthodontic treatment is tooth movement, which is optimized by light and constant forces that enhance tissue response and accelerate the process. Various techniques, including closed coil springs, closing loop arch wires, magnets, elastomeric chains, and active tiebacks, can be employed to achieve space closure in orthodontics, each providing the necessary forces to close the spaces between teeth [1]. However, each method presents unique challenges: coil springs can be difficult to keep clean, closing loop arch wires and coil springs may irritate the gingiva, and magnets are often costly, bulky, and difficult to maintain.

Elastomeric chains and active tiebacks are economical, easy to use, and relatively hygienic, providing comfort to the patient. Since the 1960s, orthodontists have employed synthetic elastomeric chains. These polyurethane polymers have replaced latex elastics for intra-arch tooth mobility. These plastic components are designed to be used in tooth rotation correction and space closure. Orthodontic elastics and elastic chains are typically composed of polyurethane elastomers and are utilized as ligatures and modules. For a variety of orthodontic purposes, these materials are frequently used as chains or single modules [2].

In order to create a complex structure of urethane linkage, polyethers or polyesters are reacted with di- or polyisocyanites in a process known as polyurethane synthesis. This procedure results in polyurethanes, which are not direct polymers of urethane. Prepolymers, which are made of linear polyester or polyether that has had its structural chain length multiplied by multiple coupling through urethane linkage, are the main component of complex polymers [3]. As polymers vary with temperature and time, they are not the ideal elastic materials.

Polymers have long-chain structures that are lightly cross-linked. This type of elasticity occurs because the polymer's macromolecular chains get distorted from their most likely shape, leading to a decrease in entropy. The restoring force that brings the polymer back to its original shape comes from this decrease in entropy. However, for the polymer to return to its original form, the movement of the chain segments must be limited. If the chains move past each other in an irreversible way, the material will be permanently deformed and will not get back to its original shape with a loss of force. When a force is applied to polymer chains, they can stretch, uncoil, or slide past one another. Chain slippage causes viscous behavior, which is slow and irreversible, while chain stretching and uncoiling cause elastic behavior, which is quick and reversible. The modules, when extended under different loading and unloading stresses, fail to return to zero elongation upon unloading, causing a permanent type of deformation [4]. Short water exposures have minimal impact on the polymers, but extended exposure to water, diluted acids, or wet heat causes them to break down, which leads to swelling and hydrolysis. The polymers get stained due to the filling of the tiny spaces in the rubber matrix by fluids and bacterial detritus in the oral cavity [5]. 

The forces applied by active tiebacks and elastomeric chains are not constant and tend to diminish with time. Prestretching the chains before insertion has been proposed as a way to counteract this force degradation and provide an active force that has already decreased to the required level for space closure. Prestretched chains, on the other hand, usually show similar degradation characteristics to non-prestretched chains. Therefore, prestretching elastomeric chains seem to have clinical advantages in orthodontic treatments [6].

It is known and has been scientifically proven that a light and continuous force should be the ideal orthodontic force exerted by any appliance for effective and rapid tooth movement. The force required to move a tooth depends on the root surface area, with the necessary magnitude of force for retraction of canines ranging from 100 g to 300-350 g. When the force applied during treatment drops below approximately 55 g, the translatory movement of the canines effectively stops [6]. Initially, elastomeric chains and active tiebacks generate sufficient force to move the teeth, but this force decreases over time. This study aimed to compare the rate of force degradation in elastomeric chains and active tiebacks with elastic modules at different time intervals. This comparison between the two techniques of canine retraction would help us to establish which method is more effective in terms of individual canine retraction when evaluated from a force degradation point of view. 

## Materials and methods

The study aimed to evaluate and compare the force degradation levels at different time intervals for canine retraction. On one side, an elastomeric chain was used, while an active tieback was applied on the opposite side. This comparison sought to determine which method was more effective in terms of better force degradation levels with respect to individual canine retraction. By examining both approaches, the study provided insights into their relative performance in force degradation. Ethical approval was obtained for this study from the Institutional Ethics Committee (IEC), with approval number IEC/VSPMDCRC/19/2022.

Study design

An analytical observational study.

Statistical analysis

Statistical analysis was performed using IBM SPSS version 21 for Windows (IBM Corp., Armonk, USA). A power analysis was established by G*Power version 3.0.1 (Heinrich-Heine-Universität Düsseldorf, Düsseldorf, Germany). A total calculated sample size of 20 subjects for a split-mouth study (20 - Group A, 20 - Group B) would yield 80% power to detect significant differences, with an effect size of 0.92 and a significance level of 0.05. Thus, 20 patients who visited the Department of Orthodontics and Dentofacial Orthopedics were included in this study based on the inclusion and exclusion criteria.

Materials used

**Figure 1:**
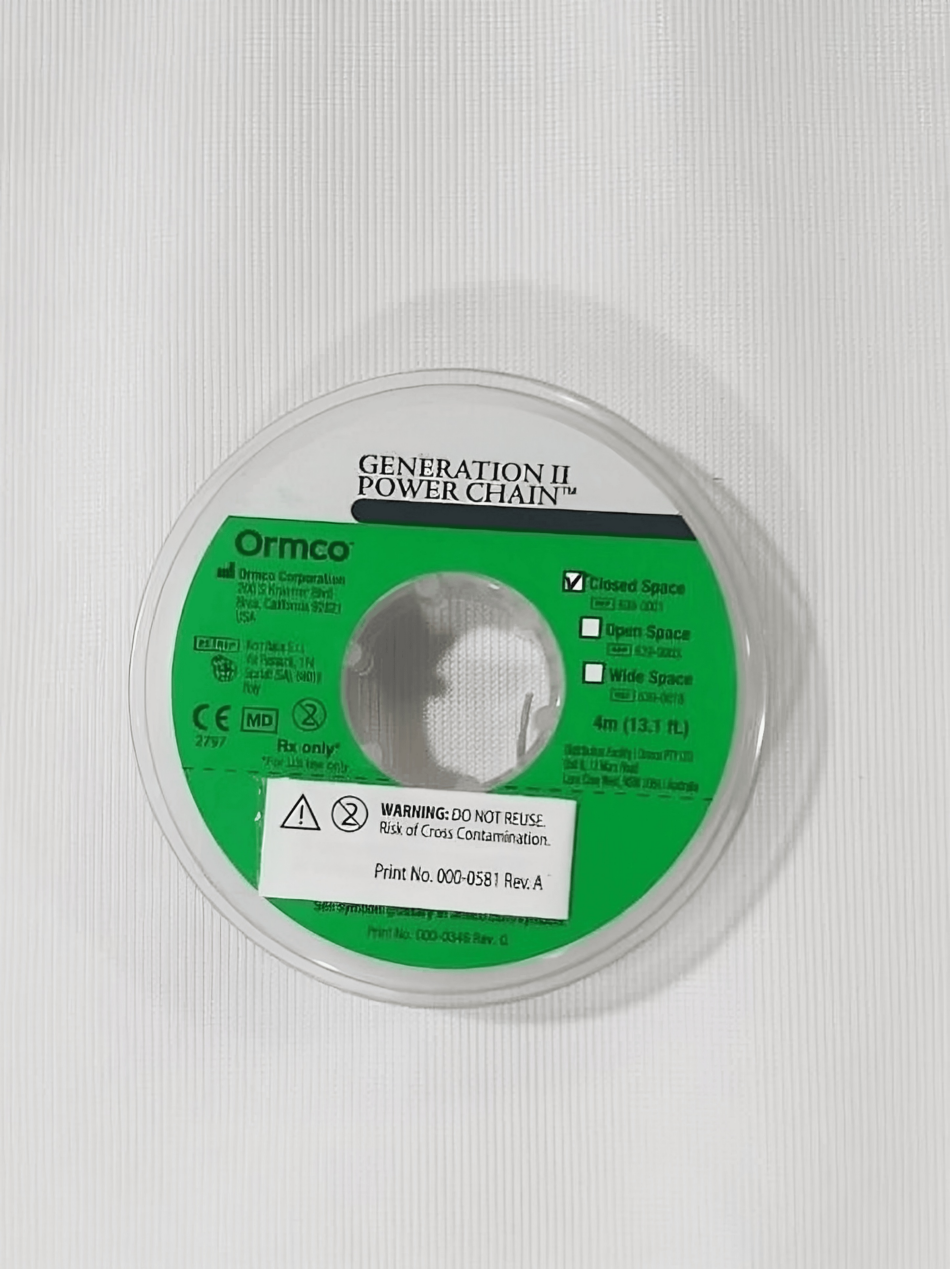
Elastomeric chain closed type (ORMCO).

**Figure 2:**
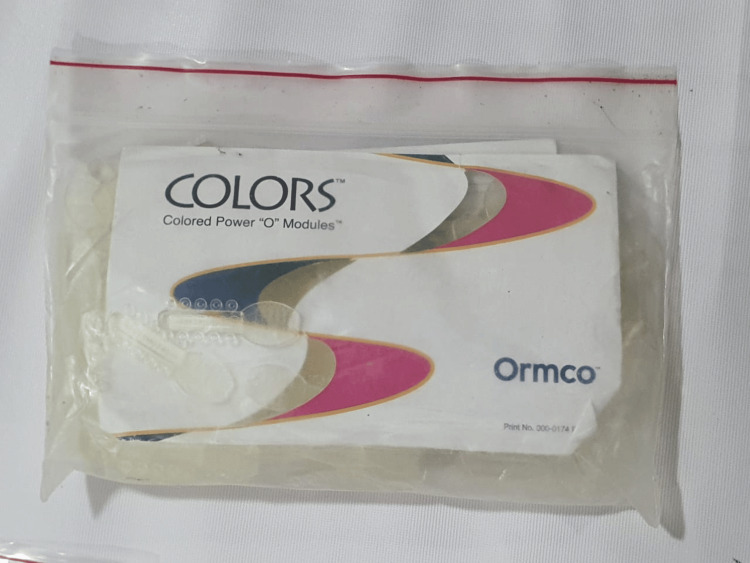
Elastic modules (ORMCO). These were used with the stainless steel ligature to make active tiebacks.

**Figure 3:**
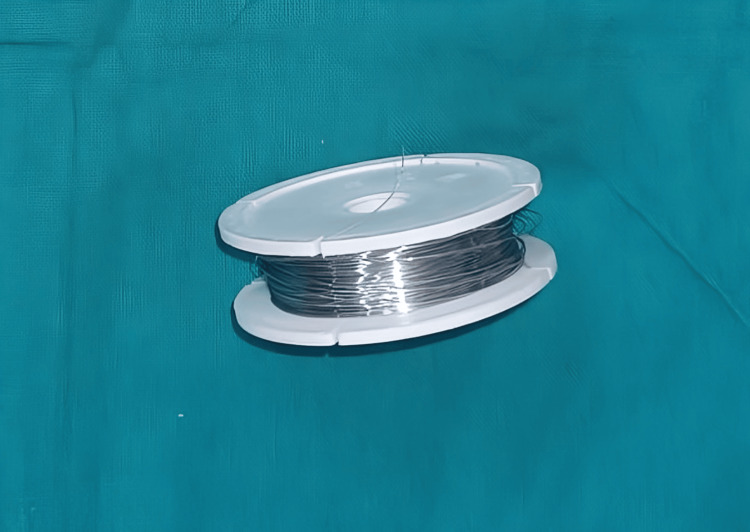
Stainless steel ligature 0.010" diameter used in the making of active tiebacks.

**Figure 4:**
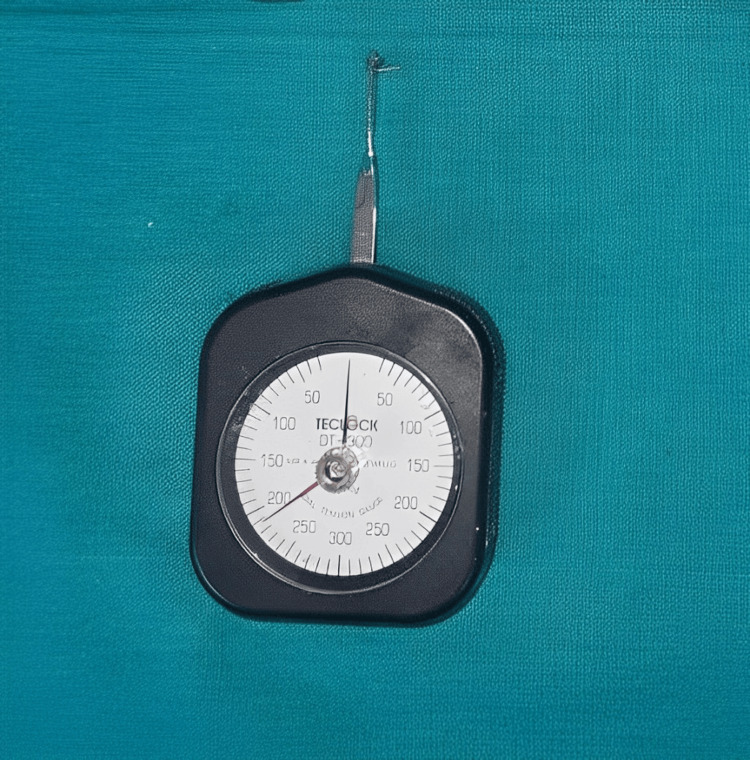
Tension gauge (TECLOCK; DT-300) for measuring the force degradation values.

An elastomeric chain closed type (ORMCO) (Figure 1), elastic modules (ORMCO) (Figure 2), stainless steel ligature (0.010” diameter) (Figure 3), and a tension gauge (TECLOCK; DT- 300) (Figure 4) were used. Active tiebacks were made with the stainless steel ligature passed through an elastic module.

Inclusion criteria

The inclusion criteria of this study were: subjects in the age group of 16 to 25 years with first premolar extraction cases where maxillary canines were planned to be individually retracted in the upper arch, subjects with no syndromes or hormonal imbalance and willingness to participate in the study, as well as physically healthy patients.

Exclusion criteria

The exclusion criteria of this study were: subjects undergoing en-masse retraction, subjects having a previous history of orthodontic treatment, subjects with a history of medical conditions, as well as subjects with poor periodontal bone support and missing teeth.

Method

**Figure 5:**
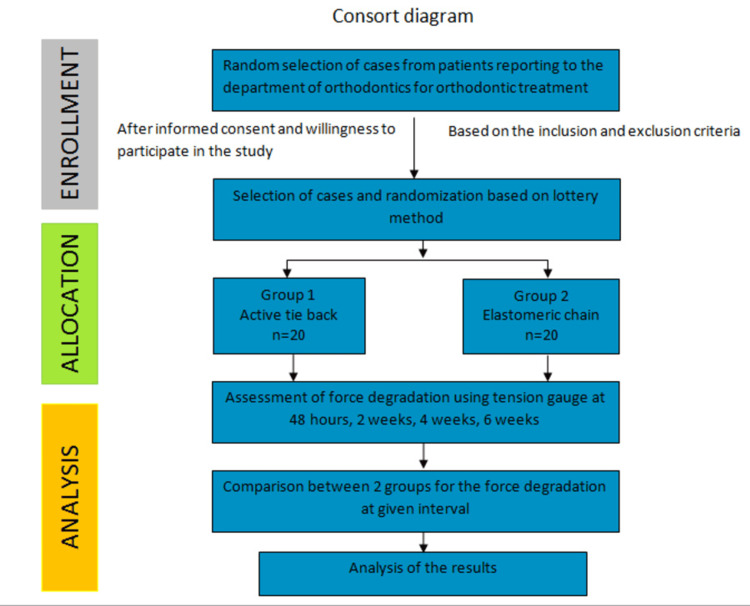
Consort diagram

Twenty subjects in the age group of 16 to 25 years who reported to the Department of Orthodontics for their orthodontic treatment were included in this study based on the inclusion and exclusion criteria and after providing informed and written consent. For patients who were less than 18 years of age, informed consent was obtained from their parents for their willingness to participate in the study. Figure 5 shows the workflow for this study. The choice of appliance for fixed orthodontic therapy was the McLaughlin-Bennett-Trevisi (MBT) 0.018" slot with canine retraction done on the 0.016 x 0.022" stainless steel arch wire [7]. Stage 1 of the treatment was leveling and alignment, after which the 0.016 x 0.022" wire was engaged. At this stage, individual canine retraction was started. Canine retraction on one side was done using the elastomeric chain, and a type 1 active tieback was used on the contralateral side. The force was kept constant at 150 g for both retraction techniques and was measured using the tension gauge. To reduce bias and randomization of the sides of the technique, a lottery system was used to decide which side should be subjected to individual canine retraction by elastomeric chain and active tieback, respectively.

Data collection

**Figure 6:**
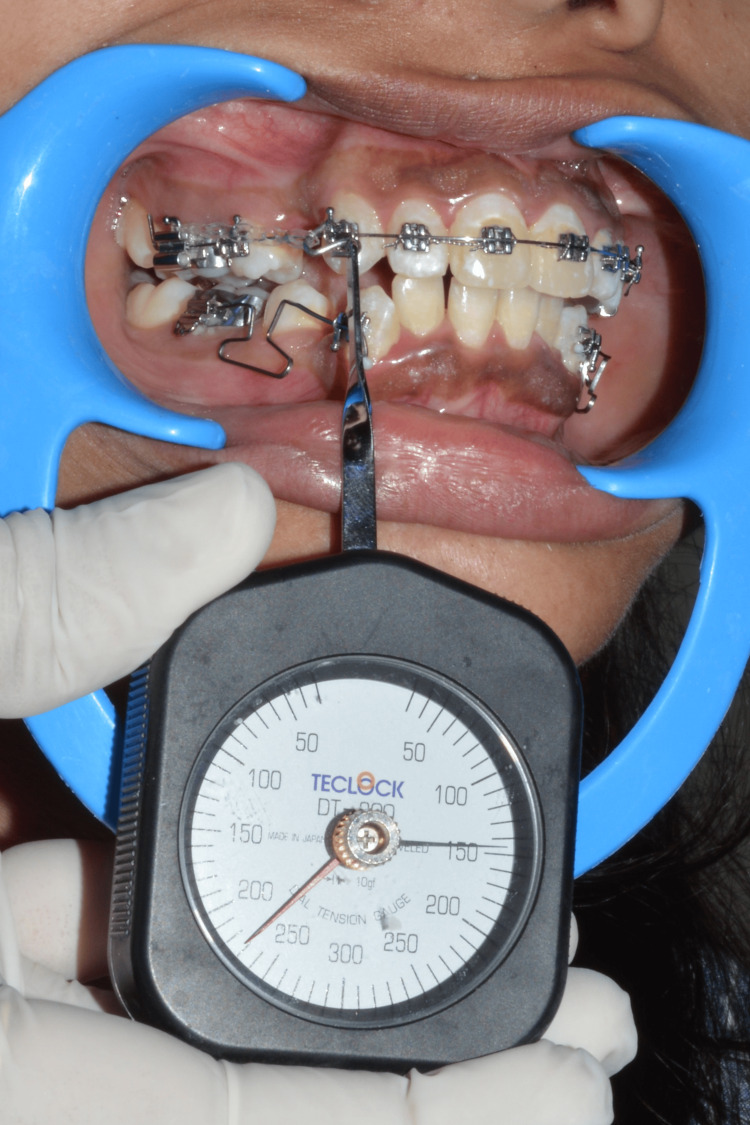
Reading of the elastomeric chain at T0 with the tension gauge. T0: Zero hours

**Figure 7:**
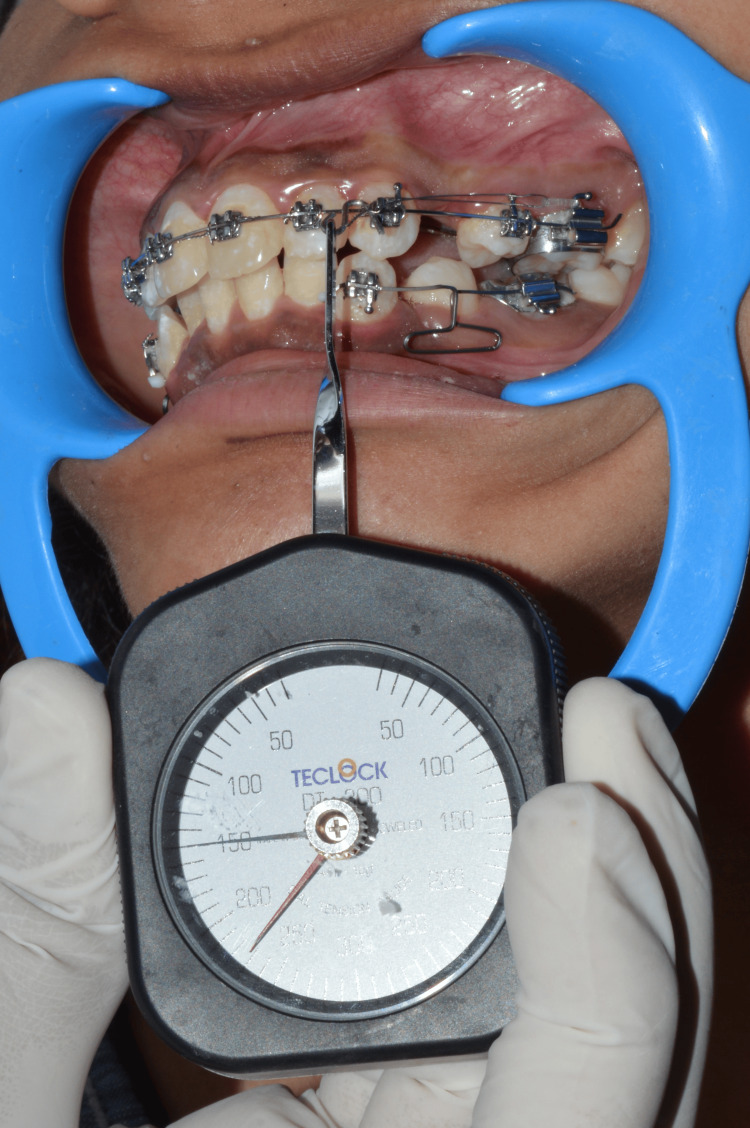
Reading of the active tieback at T0 with the tension gauge. T0: Zero hours

Data was collected at the following intervals: zero hours (T0), 48 hours (T1), two weeks (T2), four weeks (T3), and six weeks (T4). To avoid bias for force measurement, the force values were measured by two orthodontists ST and US. For a thorough examination, the gathered data was entered into a Microsoft Excel sheet (Microsoft Corp., Redmond, USA) and subjected to a statistical analysis. This documentation facilitated a detailed comparison of the effectiveness of the elastomeric chain and the active tieback methods in achieving efficient canine retraction. Details of data collection are shown in Figures 6-7. Figure 6 demonstrates the force value being measured of the elastomeric chain. Figure 7 demonstrates the force value being measured of the active tieback.

## Results

In this study, we evaluated and compared how much force degrades in elastomeric chains and active tiebacks with elastic modules at different time intervals. The readings were taken at five time intervals, as mentioned previously.

The null hypothesis of the study was that there is statistically no significant difference between the rate of force degradation of the elastomeric chain and the module in active tieback during canine retraction. The alternative hypothesis of the study was that there is a statistically significant difference between the rate of force degradation of the elastomeric chain and the module in active tieback during canine retraction.

Table 1 demonstrates the change in the rate of force degradation in the elastomeric chain group, and the obtained values were statistically compared using an analysis of variance (ANOVA) test (significant difference at p ≤ 0.05). In the elastomeric chain group, from T0, i.e., zero hours, to T4, i.e., six weeks, there was a significant decrease in the rate of force degradation.

**Table 1:** Intragroup comparison of the change in the rate of force degradation in the elastomeric chain group. Repeated measures ANOVA test. * indicates a significant difference at p ≤ 0.05. SD: Standard deviation; ANOVA: Analysis of variance

Interval	Mean	SD	F value	p-value
T0 (0 hours)	150.00	0.00	893.081	<0.001*
T1 (48 hours)	89.85	10.61		
T2 (2 weeks)	63.75	10.99		
T3 (4 weeks)	42.50	9.25		
T4 (6 weeks)	10.75	12.59		

Table 2 presents a pairwise comparison of the change in the rate of force degradation in the elastomeric chain group by using the Bonferroni test (significant difference at p ≤ 0.05). In the elastomeric chain group, there was a significant decrease in the rate of force degradation from T0 to T1, T0 to T2, T0 to T3, and T0 to T4. Similarly, there was a significant decrease in the rate of force degradation from T1 to T2, T1 to T3, and T1 to T4. The change in the rate of force degradation from T2 to T3 and T2 to T4 was also significant. Additionally, the rate of force degradation differed significantly between the last two time intervals, i.e., T3 and T4 (T3 > T4).

**Table 2:** Pairwise comparison of the change in the rate of force degradation in the elastomeric chain group. Adjustment for multiple comparisons: Bonferroni test. * indicates a significant difference at p ≤ 0.05. T0: Zero hours; T1: 48 hours; T2: Two weeks; T3: Four weeks; T4: Six weeks

Interval pair	Mean difference	p-value
T0 vs T1	60.15	<0.001*
T0 vs T2	86.25	<0.001*
T0 vs T3	107.50	<0.001*
T0 vs T4	139.25	<0.001*
T1 vs T2	26.10	<0.001*
T1 vs T3	47.35	<0.001*
T1 vs T4	79.10	<0.001*
T2 vs T3	21.25	<0.001*
T2 vs T4	53.00	<0.001*
T3 vs T4	31.75	<0.001*

Table 3 compares the change in the rate of force degradation in the elastic module in the active tieback group by using an ANOVA test (significant difference at p ≤ 0.05). In the elastic module group, from T0, i.e., zero hours, to T4, i.e., six-week time interval, there was a significant decrease in the rate of force degradation.

**Table 3:** Intragroup comparison of the change in the rate of force degradation in the elastic module in the active tieback group. Repeated measures ANOVA test. * indicates a significant difference at p ≤ 0.05. SD: Standard deviation; ANOVA: Analysis of variance

Interval	Mean	SD	F value	p-value
T0 (0 hours)	150.00	0.00	863.163	<0.001*
T1 (48 hours)	88.75	10.87		
T2 (2 weeks)	65.00	14.14		
T3 (4 weeks)	40.75	9.36		
T4 (6 weeks)	10.35	12.15		

Table 4 presents a pairwise comparison of the change in the rate of force degradation in the elastic module in the active tieback group by using the Bonferroni test (significant difference at p ≤ 0.05). In the elastic module in the active tieback group, there was a significant decrease in the rate of force degradation from T0 to T1, T0 to T2, T0 to T3, and T0 to T4. Similarly, there was a significant decrease in the rate of force degradation from T1 to T2, T1 to T3, and T1 to T4. The change in the rate of force degradation from T2 to T3 and T2 to T4 was also significant. Additionally, the rate of force degradation differed significantly between the last two time intervals, i.e., T3 and T4 (T3 > T4).

**Table 4:** Pairwise comparison of the change in the rate of force degradation in the elastic module in the active tieback group. Adjustment for multiple comparisons: Bonferroni test. * indicates a significant difference at p ≤ 0.05. T0: Zero hours; T1: 48 hours; T2: Two weeks; T3: Four weeks; T4: Six weeks

Interval pair	Mean difference	p-value
T0 vs T1	61.25	<0.001*
T0 vs T2	85.00	<0.001*
T0 vs T3	109.25	<0.001*
T0 vs T4	139.65	<0.001*
T1 vs T2	23.75	<0.001*
T1 vs T3	48.00	<0.001*
T1 vs T4	78.40	<0.001*
T2 vs T3	24.25	<0.001*
T2 vs T4	54.65	<0.001*
T3 vs T4	30.40	<0.001*

Table 5 presents an intergroup comparison of the rate of force degradation between the elastomeric chain and the elastic module in the active tieback group using an independent t-test (significant difference at p ≤ 0.05). At zero hours, the rate of force degradation between the elastomeric chain and the elastic module in the active tieback group was exactly the same. After 48 hours, the rate of force degradation was slightly greater in the elastomeric chain group as compared to the elastic module in the active tieback group; however, the difference in the rate of force degradation between the two groups after 48 hours was statistically non-significant. After two weeks, the rate of force degradation was slightly lower in the elastomeric chain group as compared to the elastic module in the active tieback group; however, the difference in the rate of force degradation between the two groups after two weeks was statistically non-significant. Again, after four and six weeks, the rate of force degradation was slightly greater in the elastomeric chain group as compared to the elastic module in the active tieback group; however, the difference in the rate of force degradation between the two groups after four and six weeks was statistically non-significant.

**Table 5:** Intergroup comparison of the rate of force degradation between the elastomeric chain and the active tieback group. Independent t-test. ATB: Active tieback; SD: Standard deviation

Interval	Elastomeric chain (n=20)		ATB group (n=20)		Difference	t-value	p-value
	Mean	SD	Mean	SD			
T0 (0 hours)	150.00	0.00	150.00	0.00	0.00	--	--
T1 (48 hours)	89.85	10.61	88.75	10.87	1.10	0.324	0.748
T2 (2 weeks)	63.75	10.99	65.00	14.14	-1.25	-0.312	0.757
T3 (4 weeks)	42.50	9.25	40.75	9.36	1.75	0.595	0.555
T4 (6 weeks)	10.75	12.59	10.35	12.15	0.40	0.102	0.919

## Discussion

This study aimed to evaluate the comparative efficacy in terms of force degradation of elastomeric chains and active tiebacks in canine retraction during fixed orthodontic therapy, thereby contributing to evidence-based orthodontic practice. When both materials were evaluated for force degradation over a period of six weeks, the result demonstrated a distinct pattern in force degradation.

This study demonstrated that the difference in the remaining force of both elastomeric chain and active tieback is not statistically significant. In our study, the force degradation of the active tieback after 48 hours was 40.83% and that of the elastomeric chain was 40.10% of the initial force. The greatest amount of force degradation is 20-50% depending on the study and chain type, within 24 hours, followed by a considerably slower rate of degradation over the next four weeks, providing an average degradation of 50-85% [8]. Studies have shown a significant amount (42-75%) of force degradation within the first 24 hours of elastomeric chain application [9-12]. Andreasen and Bishara in 1970 [9] measured elastic forces with materials submerged in water and materials submerged in saliva at a constant 37°C temperature for three weeks with a correx gauge and concluded that 74% of the initial force of the chains was lost. Wong et al. in 1976 evaluated the changes in force and physical properties that occurred in the mouth with elastomeric materials by stretching the latex elastomer over a 21-day period. The findings of Wong et al.'s study showed that for chains stretched to a continuous length over a 24-hour period, just 50% of the initial force persisted [10]. Taloumis et al. [11] in 1997 assessed the force decay of commercially available, moulded, grey elastomeric ligatures from seven different producers and demonstrated a 53% to 68% force degradation in the first 24 hours. Grassi et al. [12] in 2001 measured elastic force decay at one, seven, 14, and 21 days and observed that elastomeric chains reduced their elastic rebound to 50-70% of the initial rate within 24 hours. But, in the above-mentioned studies, the value was not measured against active tieback, which is very commonly used. In our study, the force degradation after four weeks was 70%, which is similar to the findings of Yang et al. who observed that the force degradation of four-unit elastomeric chains after four weeks was 64.8% [13]. 

Lu et al. [14] in 1993 observed that the larger the force, the greater the initial force degradation. However, in their investigation, the chains were stretched to the point where their elastic limits were reached, which increased the force degradation. These findings contradict those of De Genova et al. [15] who claimed that modules with larger initial forces saw less force degradation. In this study, the initial forces of the groups were the same, and smaller forces were applied, i.e., 150 g. Others indicated that there was no relationship between the initial force, the amount of force decay, and the amount of space closure [16,17]. Our study was designed to evaluate elastic ligatures in a tieback setup. Previous studies have not evaluated and compared both techniques.

Storey and Smith [18] and Reitan [19] concluded that 100 to 250 g of force is optimum for canine retraction, whereas Hixon [20] reported that 350 g of force was optimum for canine retraction. In this study, a light, continuous force was used for canine retraction. Thus, 150 g of force was applied for effective canine retraction based on previously established values by Storey and Smith [18]. It is very challenging to apply constant force with any system; therefore, orthodontists must choose a system that provides a more effective force. Our study findings suggested that tieback and elastomeric chains both have similar force degradation and are suitable approaches for space closure. A study done by Bennett and McLaughlin [21] proposed that tieback was a suitable space closure mechanism since elastomeric chains produced inconsistent forces, were difficult to maintain, and occasionally broke apart.

An increase in force degradation in both groups was seen in the last two weeks. This phenomenon can be attributed to the diminution of intermolecular forces within the materials due to exposure to saliva, which could involve various enzymatic actions and cumulative degradation effects.

In this study, the residual force in both groups after four weeks was around 25% (i.e., about 40 g). This finding is in agreement with Grassi et al. [12] who observed that after three weeks the elastomeric chain's residual force ranged from 20% to 40% of its initial activation. After four weeks, however, the elastomeric chains' remaining force was less than the ideal force. According to Josell [6], the canine movement ceases at 55 g or less force. Thus, in our study, we observed that the force level of the elastomeric chain was reduced to such a level that further retraction of the canine was not possible.

Considering the aging of elastic materials and observed findings of our study, it is suggested that replacing the elastomeric chains and tiebacks before four weeks may be clinically required. This is consistent with the study by Bennet [21] and Manohar et al. [1], which recommended replacing tiebacks every four to six weeks. As observed in this study, the forces maintained after four weeks did not become 0 g. Taloumis et al.'s [11] study findings are consistent with our study findings as all of the elastomeric chains and ligatures examined had a residual force after four weeks.

In this study, the percentage of residual force in tiebacks and chains was around 7% after six weeks. This was consistent with the research by Samuels et al. [22] who observed that in certain situations forces produced by elastic modules even decreased to zero after five to eight weeks. 

When teeth experience light, sustained pressure, the periodontal ligament (PDL) gets partially compressed on the pressure side and stretched on the tension side [23]. This process causes the teeth to move within their sockets. After applying force, this histophysiological phenomenon happens within hours. When force levels are kept constant and low, frontal resorption occurs, which allows teeth to migrate gradually. Increased force levels cause undermining resorption since the neighboring bone doesn't break down as quickly, causing tooth movement that alternates between periods of stasis and movement. Thus, after 48 hours, the initial force degraded by 40% and then a low and continuous force was maintained for canine retraction.

Limitations and future scope

The study included a focused group of 20 patients, providing valuable insights, though further research with a larger population could help confirm and expand these findings. In this study, commonly used older materials were employed for canine retraction. While these materials are widely accepted, incorporating more efficient and advanced materials in future research could provide better results and optimize treatment efficiency.

## Conclusions

To conclude, both groups - elastomeric chain and active tieback - experienced rapid force decay within the first 48 hours. The force decay was similar in both groups. After 48 hours, a more constant and stable orthodontic force was retained for tooth movement. As time passed, the force in both groups gradually decreased. After four weeks, the force dropped to a level where it eventually became too low to move the teeth any further, and thus the tooth movement ceased. Both elastomeric chain and tieback are acceptable methods for space closure in orthodontics, but they should be changed every four weeks to maintain a constant force for continuous retraction.
